# Supplementary material for: Selective antibiofilm properties and biocompatibility of nano-ZnO and nano-ZnO/Ag coated surfaces
Source: Sci Rep. 2020 Aug 10;10:13478. doi: 10.1038/s41598-020-70169-w (PMC7417576; doi:10.1038/s41598-020-70169-w)
Supplement: Supplementary file 1 — Supplementary information [file 41598_2020_70169_MOESM1_ESM.pdf]

Selective antibiofilm properties and biocompatibility of nano-ZnO and nano-ZnO/Ag coated surfaces

M. Rosenberg*[1, 2], M. Visnapuu[3], H. Vija[1], V. Kisand[3], K. Kasemets[1], A. Kahru[1, 4], A. Ivask[1, 5]

[1] Laboratory of Environmental Toxicology, National Institute of Chemical Physics and Biophysics, Tallinn, Estonia
[2] Department of Chemistry and Biotechnology, Tallinn University of Technology, Tallinn, Estonia
[3] Institute of Physics, University of Tartu, Tartu, Estonia
[4] Estonian Academy of Sciences, Tallinn, Estonia
[5] Institute of Molecular and Cell Biology, University of Tartu, Tartu, Estonia

* rosenbergmerilin@gmail.com

Supplementary figures

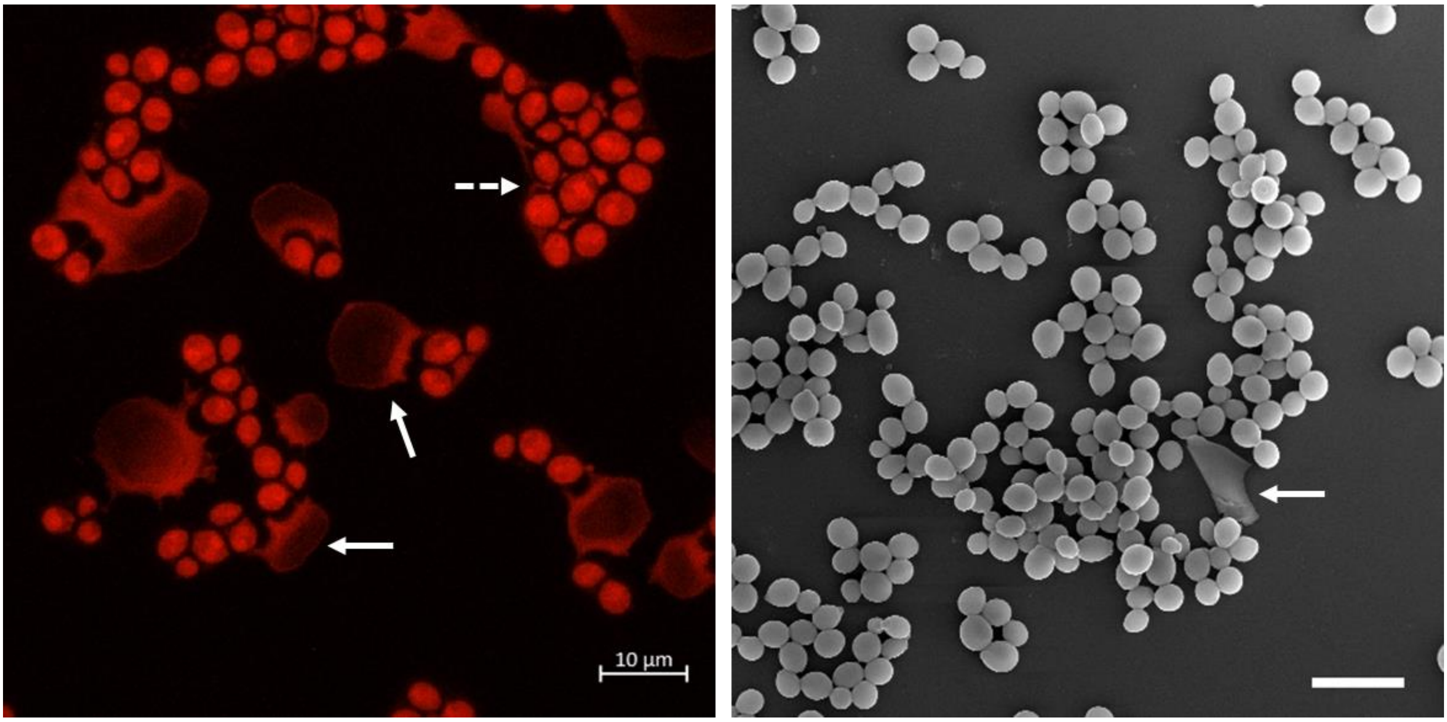

Supplementary Figure 1. 48 h *C. albicans* biofilm on sparse ZnO/Ag surface in growth medium: CLSM maximal orthogonal projection with cell walls stained red with Congo red (left) and SEM image (right). White arrows indicate flattened dead “ghost cells” that occur in patches on nano-ZnO/Ag surfaces and seem to act as a carrier for biofilm cells (dotted arrow). Most “ghost cells” are laterally detached from neighboring cells during several liquid manipulation steps in the fixing protocol and are mostly lost in SEM images. Scale bars represent 10 µm.

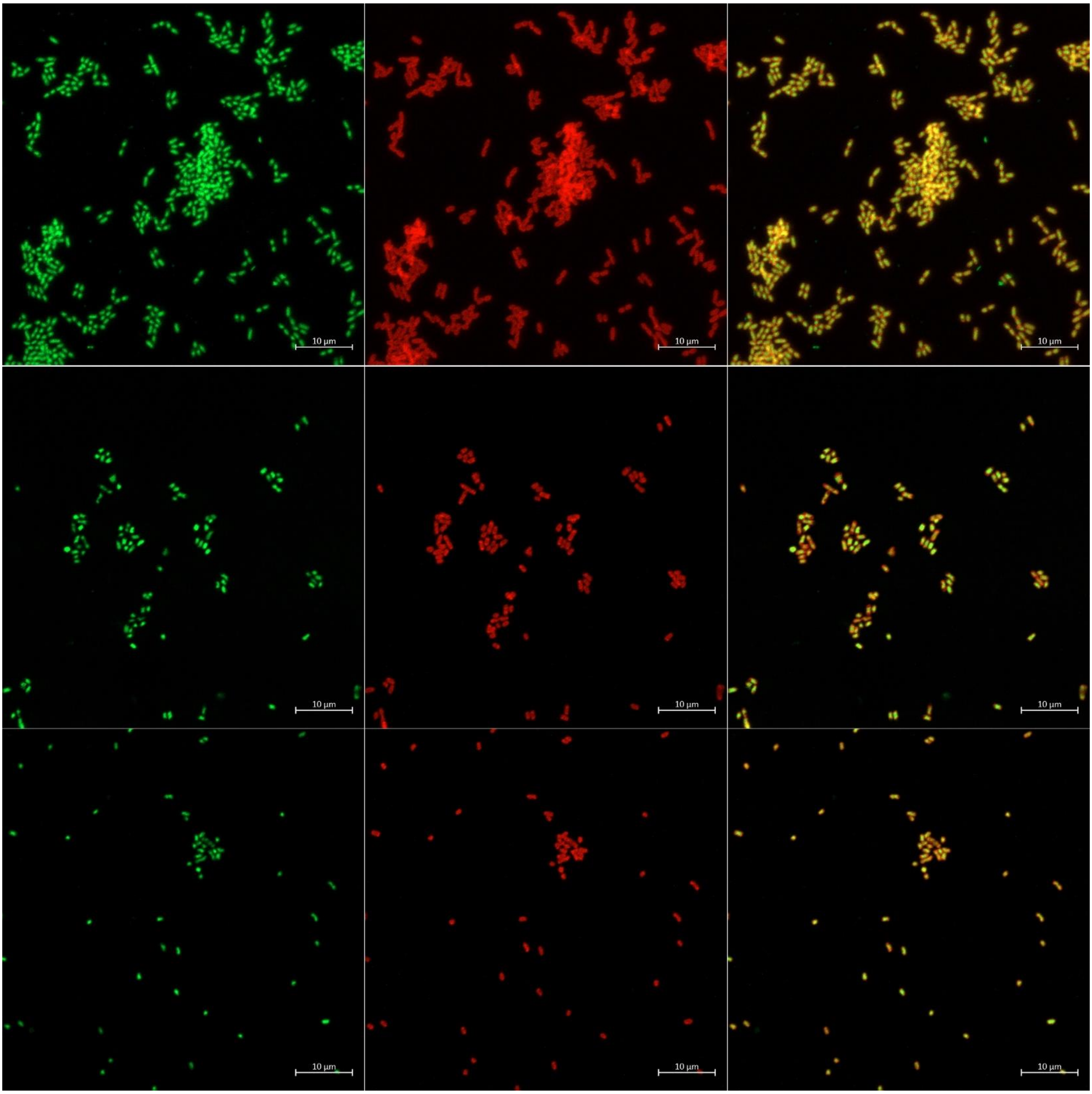

Supplementary Figure 2. Maximal orthogonal projections of representative CLSM images of fixed 48 h oligotrophic *E. coli* biofilm on uncoated surface (upper panel), sparse nano-ZnO surface (middle panel) and dense nano-ZnO (bottom panel). DNA/RNA stained with Syto9 (green channel, left column) and surface-associated amyloid fibers stained with Congo Red (red channel, middle column). Combined channel view in right column. Scale bars represent 10 µm.

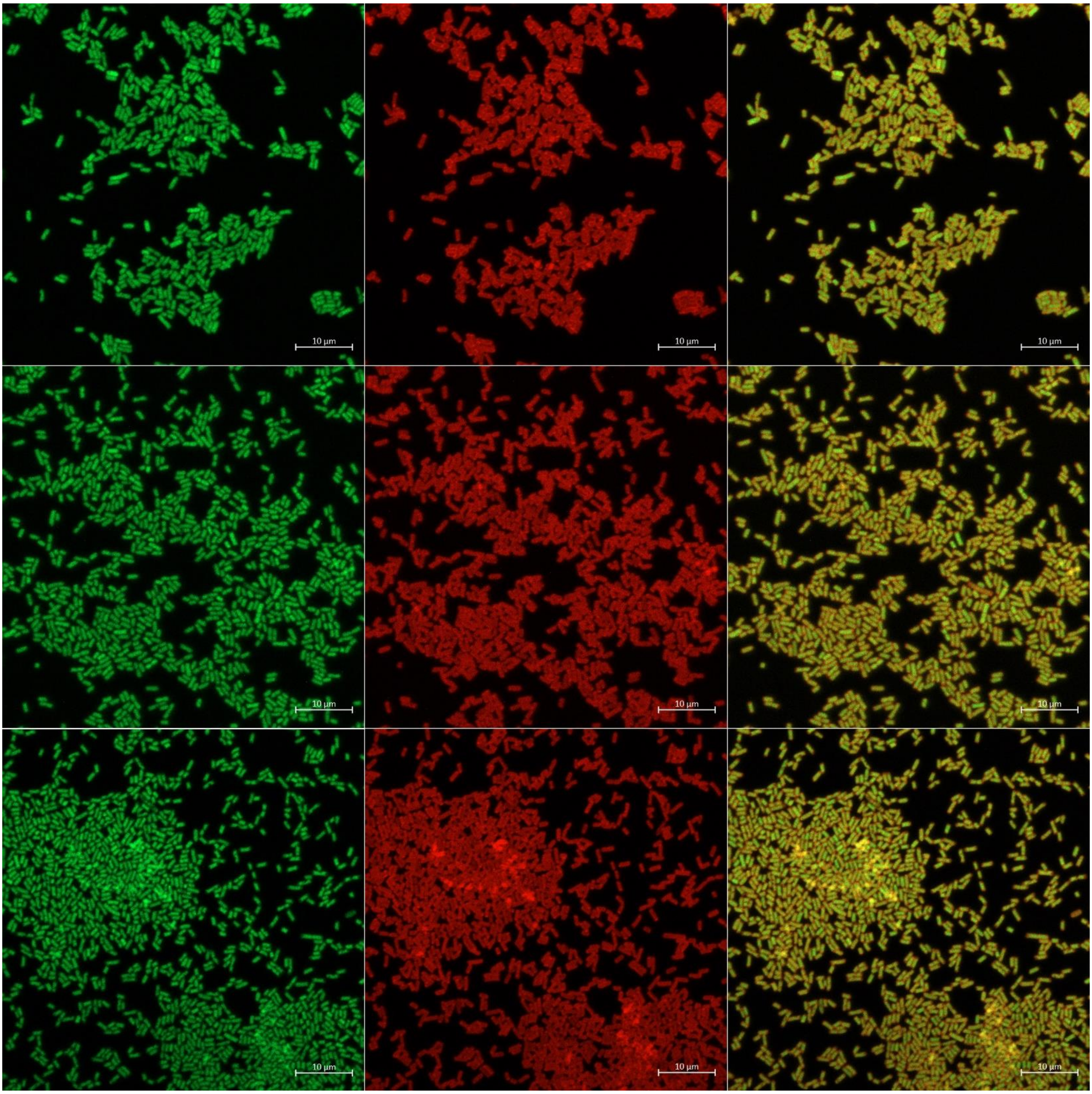

Supplementary Figure 3. Maximal orthogonal projections of representative CLSM images of fixed 48 h growth medium *E. coli* biofilm on uncoated surface (upper panel), sparse nano-ZnO surface (middle panel) and dense nano-ZnO (bottom panel). DNA/RNA stained with Syto9 (green channel, left column) and surface-associated amyloid fibers stained with Congo Red (red channel, middle column). Combined channel view in right column. Scale bars represent 10 µm.

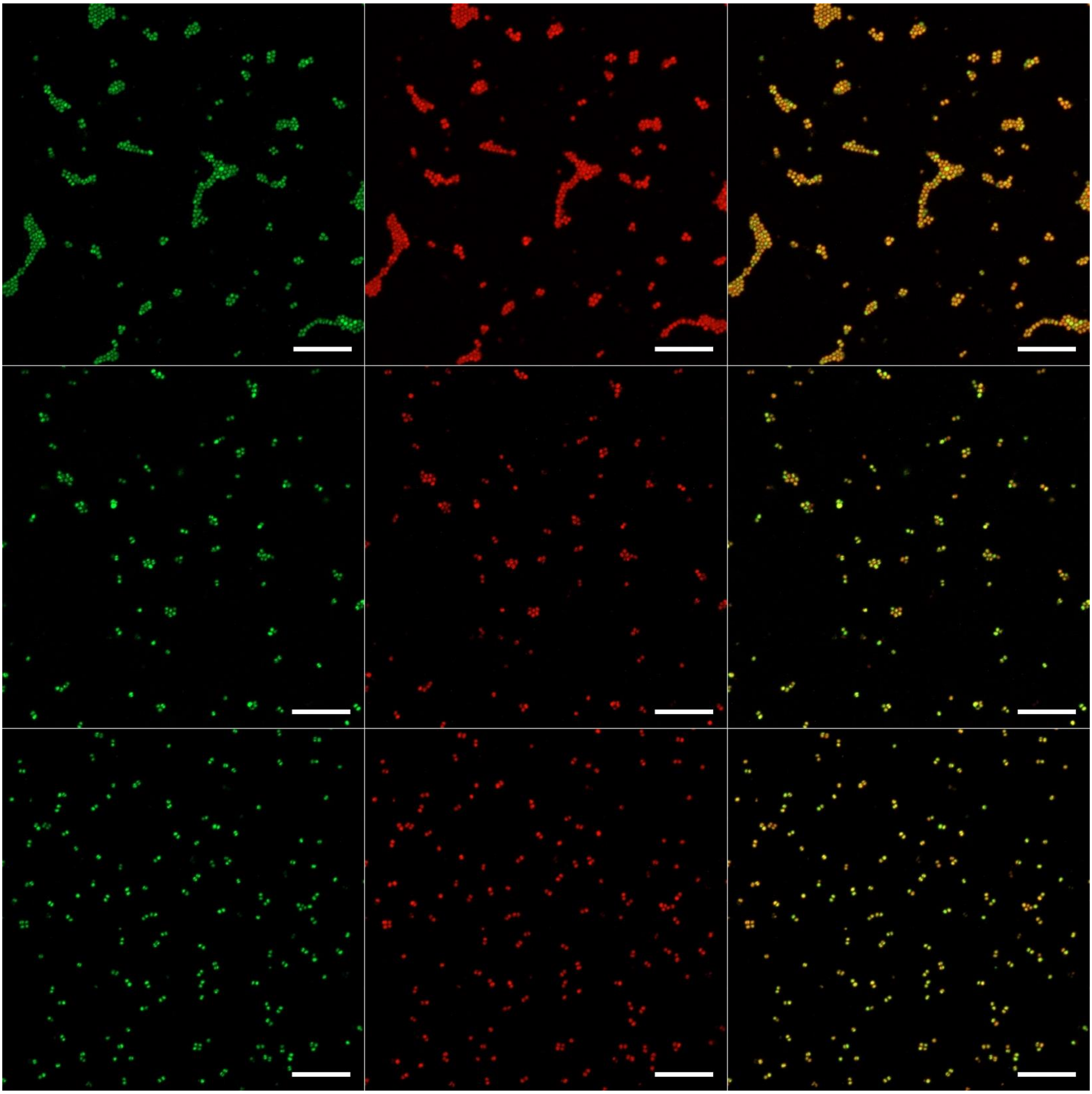

Supplementary Figure 4. Maximal orthogonal projections of representative CLSM images of fixed 48 h oligotrophic *S. aureus* biofilm on uncoated surface (upper panel), sparse nano-ZnO surface (middle panel) and dense nano-ZnO (bottom panel). DNA/RNA stained with Syto9 (green channel, left column) and surface-associated amyloid fibers stained with Congo Red (red channel, middle column). Combined channel view in right column. Scale bars represent 10 µm.

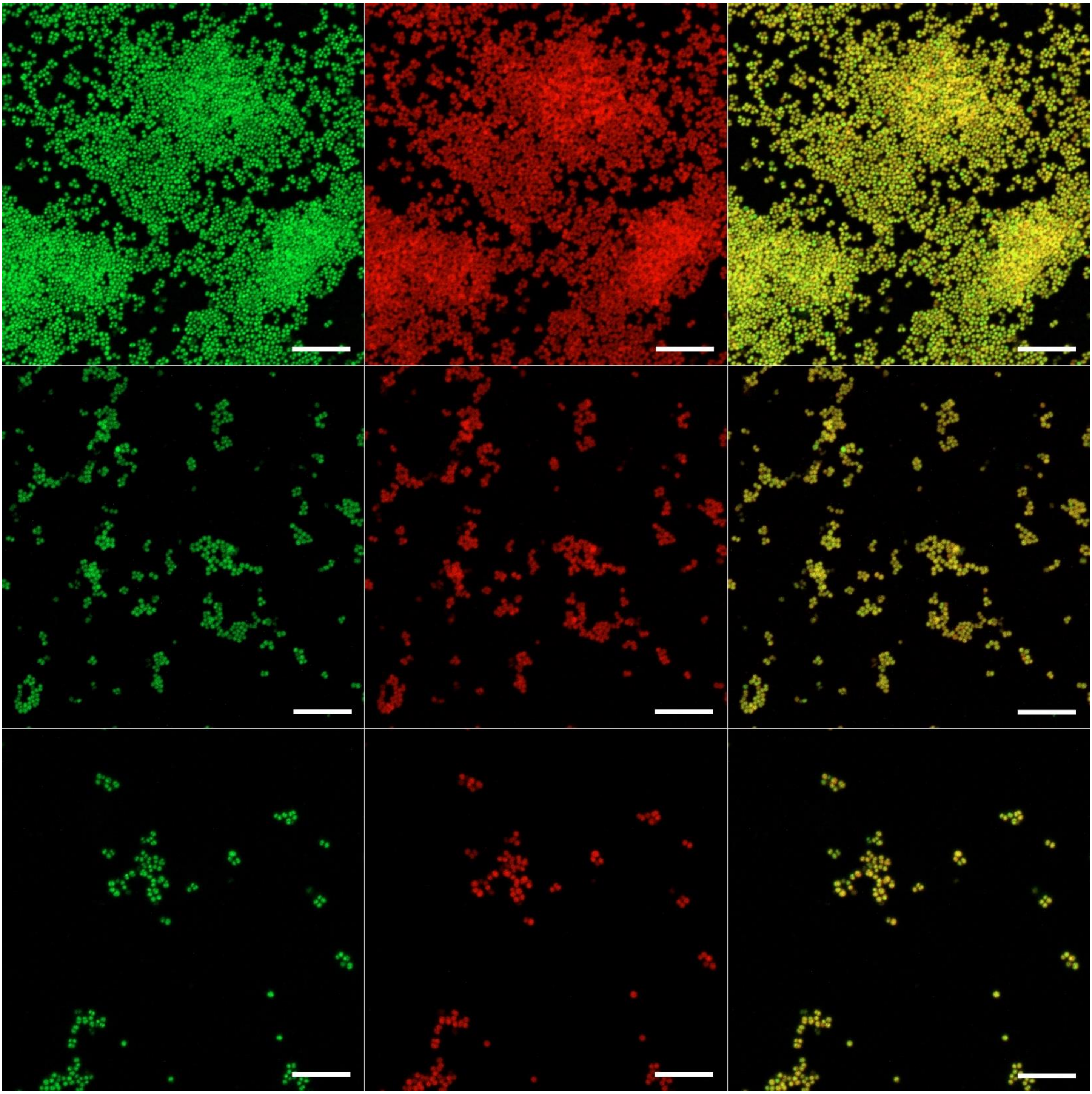

Supplementary Figure 5. Maximal orthogonal projections of representative CLSM images of fixed 48 h growth medium *S. aureus* biofilm on uncoated surface (upper panel), sparse nano-ZnO surface (middle panel) and dense nano-ZnO (bottom panel). DNA/RNA stained with Syto9 (green channel, left column) and surface-associated amyloid fibers stained with Congo Red (red channel, middle column). Combined channel view in right column. Scale bars represent 10 µm.

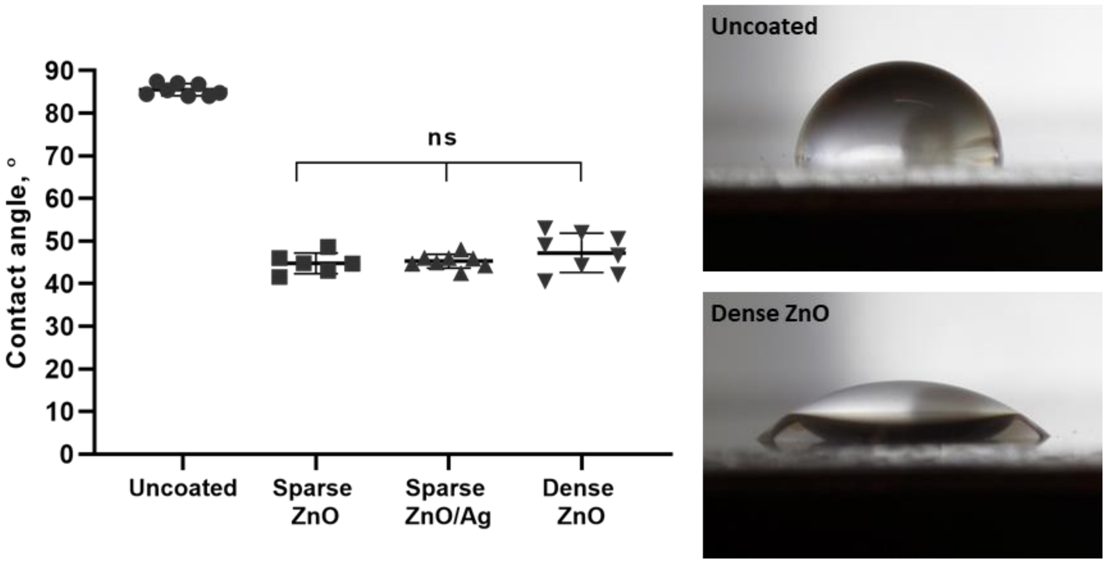

Supplementary Figure 6. Water contact angles of uncoated, nano-ZnO and nano-ZnO/Ag surfaces. The angles were measured from photos taken 5 sec after pipetting 5 µL of water to each surface, using Contact Angle plugin (author Marco Brugnara) in ImageJ software [1]. ns – not statistically significant

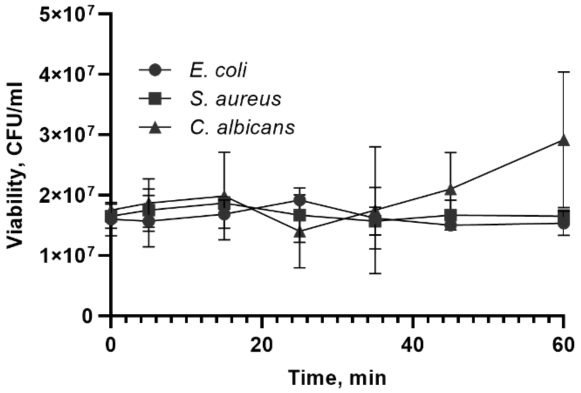

Supplementary Figure 7. Microbial viability in biofilm harvesting medium (0.5x SCDLP with 1.5M NaCl, Table 1). 15 ml microbial was vortexed for 30 sec, sonicated for 0-60 min, vortexed again for 30 sec, serially diluted and plated for counting. Harvesting protocol in 0.5x SCDLP with high salt concentration did not significantly affect microbial viability after 15 min sonication compared to the initial suspension (P≥0.05).

Supplementary Table 1. Zn and Ag content in the nano-ZnO and nano-ZnO/Ag surfaces; theoretical maximum release into 5 ml microbial test medium. ND denotes "not detected".

Surface coating	Zn μg/cm^2	SD	Max Zn release into 5 ml, μg/ml	SD	Ag ng/cm2	SD	Max Ag release into 5 ml, ng/ml	SD
Uncoated	0.02	0.00	0.01	0.00	ND	ND	ND	ND
Sparse nano-ZnO	2.12	0.12	1.36	0.08	ND	ND	ND	ND
Sparse nano-ZnO/Ag	2.39	0.10	1.54	0.07	23.56	3.42	15.27	2.21
Dense nano-ZnO	18.00	0.70	11.65	0.45	ND	ND	ND	ND

References

[1] C. A. Schneider, W. S. Rasband, and K. W. Eliceiri, "NIH Image to ImageJ: 25 years of image analysis," *Nat Methods*, vol. 9, no. 7, pp. 671–675, Jul. 2012, doi: 10.1038/nmeth.2089.
